# Resting State Functional Connectivity Biomarkers of Treatment Response in Mood Disorders: A Review

**DOI:** 10.3389/fpsyt.2021.565136

**Published:** 2021-03-26

**Authors:** Joseph J. Taylor, Hatice Guncu Kurt, Amit Anand

**Affiliations:** ^1^Center for Brain Circuit Therapeutics, Brigham and Women's Hospital, Harvard Medical School, Boston, MA, United States; ^2^Center for Behavioral Health, Cleveland Clinic, Cleveland, OH, United States

**Keywords:** resting state functional connectivity, fMRI, depression, bipolar disorder, mood disorder, biomarker, treatment response, neuroimaging

## Abstract

There are currently no validated treatment biomarkers in psychiatry. Resting State Functional Connectivity (RSFC) is a popular method for investigating the neural correlates of mood disorders, but the breadth of the field makes it difficult to assess progress toward treatment response biomarkers. In this review, we followed general PRISMA guidelines to evaluate the evidence base for mood disorder treatment biomarkers across diagnoses, brain network models, and treatment modalities. We hypothesized that no treatment biomarker would be validated across these domains or with independent datasets. Results are organized, interpreted, and discussed in the context of four popular analytic techniques: (1) reference region (seed-based) analysis, (2) independent component analysis, (3) graph theory analysis, and (4) other methods. Cortico-limbic connectivity is implicated across studies, but there is no single biomarker that spans analyses or that has been replicated in multiple independent datasets. We discuss RSFC limitations and future directions in biomarker development.

## Introduction

Psychiatric disorders are currently defined by symptom clusters in the Diagnostic and Statistical Manual of Mental Health Disorders, 5th Edition (DSM-5) (1). These clinical constructs are fairly reliable (2–4), but they lack biological validity. Research Domain Criteria (RDoC) aims to address this issue by providing a neuroscientific framework in which to study psychiatric symptoms independent of DSM classifications (5–9). Attempts to create new clinical phenotypes by mapping symptoms onto RDoC constructs have had limited success (10), highlighting the need to identify biological substrates and biomarkers.

A biomarker is a “characteristic that is measured as an indicator of normal biological processes, pathogenic processes, or responses to an exposure or intervention, including therapeutic interventions” (11). As such, biomarkers can represent clinically relevant intermediate outcomes or endpoints that are difficult to measure. Several branches of medicine have used biomarkers to identify pathology in presymptomatic or asymptomatic individuals, elucidate treatment mechanisms of action, predict or monitor treatment response, improve existing treatments, and develop new treatments (12). There are currently no established biomarkers in psychiatry, a field with few measurements besides scales for reported or observable symptoms.

Neuroimaging is a critical tool for developing psychiatric biomarkers (6). Traditional functional neuroimaging examines the spatial and temporal characteristics of blood oxygen level-dependent (BOLD) signal during alternating blocks of task and rest. What happens during rest is essentially treated as noise, a spontaneous signal drift to be subtracted from task block data. Newer paradigms question this traditional perspective on signal vs. noise, especially since task-based BOLD signal only accounts for a fraction of overall neural metabolism (13). Connectivity analyses of rest blocks has revealed spatial and temporal patterns that effectively launched the field of resting state functional connectivity (RSFC) (13, 14).

The ability of RSFC to identify known connections between brain regions has been empirically validated (15–18). Functional connectivity does not necessarily imply anatomical connectivity, although this relationship can be useful to explore if present (19–21). There are several advantages to RSFC vs. task-based activation, including simpler data acquisition and greater capacity to detect individual- and group-level differences (22). Despite these advantages, RSFC is inherently noisier because signal fluctuations are highly dynamic yet low in amplitude (23). There are also issues such as scanner drift, motion artifact, and limited signal-to-noise in ventral brain regions that are prominent in but not unique to RSFC (24–29). These factors make it challenging to measure intra- and inter-scan reliability within or between studies. Furthermore, the wide variety of RSFC analytic techniques complicates interpretation between studies.

The present study is a review of RSFC biomarkers of treatment response in mood disorders, which are prevalent and often debilitating conditions associated with chronic illnesses, suicide, and all-cause premature death (30–40). There are effective treatments for mood disorders, but at least one-third of patients do not remit despite multiple treatment trials (41, 42). Furthermore, little is known about treatment mechanisms of action, making it difficult to select specific treatments for specific symptom profiles or patients (5, 7, 9, 43–45). Several existing reviews have successfully highlighted task-based and RSFC biomarkers of mood disorders (46–55). We aim to expand this knowledge base by focusing on the evidence for biomarkers of treatment response across diagnoses, theoretical brain network models, and treatment modality. To this end, our review is organized around the following primary analytic techniques: (1) reference region (seed-based) analysis, (2) independent component analysis (ICA), (3) graph theory analysis, and (4) other methods. We hypothesized that there will be no single treatment biomarker validated for mood disorders across diagnoses, models, treatment modalities, and independent datasets.

## Methods

We followed general Preferred Reporting Items for Systematic Reviews and Meta-Analyses (PRISMA) guidelines (56, 57) in this review of TSFC biomarkers of treatment response in mood disorders. HGK searched PubMed for studies with various combinations of the following terms: “resting state,” “functional connectivity,” “fMRI,” “treatment effects,” “neuroimaging biomarkers,” “mood disorders,” “depression,” and “bipolar disorder.” During the first quarter of 2020, HGK identified candidate abstracts of studies reporting RSFC changes following treatment as well as studies attempting to predict treatment response based on RSFC. Studies meeting these criteria were reviewed by JJT, HGK, and AA. Studies were excluded if they were not written in English, if they did not include treatment, or if they reported RSFC changes in healthy participants. Results are organized by RSFC analysis method. See Supplementary Material for a table summarizing the main studies discussed in the text.

## Results

### Reference Region (Seed-Based) Analysis

Reference region analysis, or seed-based analysis, involves assessing the time series correlation between an *a priori* region of interest (ROI) and all other voxels in the brain (17). Whole-brain, voxel-wise functional connectivity maps of co-variance with the seed region are usually generated from general linear model analysis (58). Like many other FSFC analyses, seed-based analysis assumes that temporal correlation between two or more regions implies a functional connection between them, potentially revealing a brain circuit or network (59). Reference region analysis is considered univariate because voxelwise data are regressed against the broader model in an independent manner (58).

RSFC between two brain regions is typically bidirectional. The following section is organized based on which ROI connectivity was defined as the primary measure in each particular study.

#### Anterior Cingulate Cortex

The anterior cingulate cortex (ACC) is of particular interest in mood disorders. It is typically divided into dorsal/pregenual and rostral/subgenual regions based on cytoarchitecture, connectivity, and putative function in healthy controls.

##### Dorsal/Pregenual Anterior Cingulate Cortex

The dorsal/pregenual region has been consistently implicated in conflict resolution (60) and in several processes associated with depression (18, 61, 62). RSFC between dorsal/pregenual ACC and limbic regions such as amygdala, striatum, and medial thalamus is decreased in depression (18), and this connectivity increases after successful treatment with sertraline (63). Clinical symptom improvement correlates with increased dorsal/pregenual ACC-amygdala connectivity and decreased amygdala activation (64). ACC connectivity changes have also been documented after successful electroconvulsive therapy (ECT) for unipolar and bipolar depression, with specific changes in connectivity to orbitofrontal cortex, caudate, dorsolateral prefrontal cortex, and posterior cingulate cortex (65).

The data on whether dorsal/pregenual connectivity can predict treatment response are limited and mixed. One study of late-life depression reported that decreased RSFC between dorsal/pregenual ACC and other regions of the cognitive control network predicted low remission rates after 12 weeks of escitalopram (61). In a different study, increased RSFC of the dorsal/pregenual ACC to the cognitive control network predicted non-response to 12 weeks of escitalopram, duloxetine, or venlafaxine (62). These conflicting reports are difficult to reconcile, and more data are needed.

##### Rostral/Subgenual Anterior Cingulate Cortex

The rostral/subgenual ACC, which encompasses Brodmann area 25 and is also known as subcallosal ACC, has been frequently implicated in the pathophysiology of major depression (66, 67). Rostral/subgenual ACC is generally found to be anticorrelated with the dorsal/pregenual ACC. In other words, rostral/subgenual ACC RSFC to limbic regions is increased in depression (68, 69).

Several studies have explored whether RSFC connectivity of the rostral/subgenual ACC can predict treatment outcomes across treatment modalities (70–78). One study found a positive correlation between baseline RSFC of right rostral/subgenual ACC to right dorsolateral prefrontal cortex (DLPFC) and improvement in depressive symptoms following group cognitive-behavioral therapy (74). A different study found a negative correlation between depression improvement and baseline RSFC between left rostral/subgenual ACC and the broader left ACC after 8 weeks of monotherapy with bupropion, escitalopram, or aripiprazole (70).

Some studies have used RSFC of rostral/subgenual ACC to explain differential outcomes following cognitive behavioral therapy vs. medication. In a study of 122 patients with major depressive disorder, positive RSFC of rostral/subgenual ACC with left anterior ventrolateral prefrontal cortex, insula, dorsal midbrain, and left ventromedial prefrontal cortex was associated with remission after CBT and non-response to escitalopram or duloxetine monotherapy. Negative connectivity was associated with the opposite response outcome. Regardless of treatment modality, response was positively correlated with rostral/subgenual ACC RSFC to right post-central gyrus and negatively correlated with rostral/subgenual ACC RSFC to right superior frontal gyrus. By contrast, remission was negatively correlated with rostral/subgenual ACC RSFC to right precentral gyrus and right posterior putamen (71).

RSFC of rostral/subgenual ACC has also been explored in psychedelics and interventional psychiatry. Psilocybin was shown to increase rostral/subgenual ACC RSFC with posterior cingulate cortex and precuneous, but this effect was not correlated with clinical improvement (79). By contrast, increased RSFC between rostral/subgenual ACC and right lateral prefrontal cortex was positively correlated with response to a single ketamine infusion (78).

One of the most robust and convincing lines of research investigating rostral/subgenual ACC is its relationship to transcranial magnetic stimulation (TMS) response. Numerous studies have used baseline rostral/subgenual ACC RSFC to predict or optimize TMS response, although the network itself varies slightly between studies. For example, DLPFC regions (specifically within Brodmann Area 46) that are most anticorrelated to rostral/subgenual ACC tend to be the most effective TMS treatment targets (73). Other studies have taken slight variations on this network, with one showing that TMS responders had stronger baseline anticorrelation between rostral/subgenual ACC and medial and left superior frontal gyrus (specifically Brodmann Area 10) (77). A different group reported that baseline rostral/subgenual ACC RSFC with ventromedial prefrontal cortex, dorsomedial prefrontal cortex, dorsal/pregenual ACC and posterior cingulate cortex predicted TMS response (80). The role of the rostral/subgenual ACC seems to be preserved even if the TMS target or patient population changes. One study of patients with unipolar or bipolar depression showed that response to dorsomedial prefrontal cortex TMS was associated with positive RSFC between rostral/subgenual ACC and DLPFC and negative RSFC between rostral/subgenual ACC and insula, putamen, parahippocampus, and amygdala (76). The rostral/subgenual ACC RSFC also seems to be implicated in accelerated TMS studies as well, with one study showing that responders had stronger rostral/subgenual ACC correlation with medial orbitofrontal cortex after treatment (77).

In the context of these TMS results, it is important to note that there is some convergent evidence for rostral/subgenual ACC across neuromodulation modalities. RSFC of rostral/subgenual ACC has been used to predict ECT response (72), and BA 25 within this region is the primary target for deep brain stimulation for treatment-resistant depression (81–87).

#### Orbitofrontal Cortex

Orbitofrontal cortex is often implicated in RSFC changes from other seed regions, but there is not a robust literature for it as a primary seed region in mood disorder treatment response. In one study, increased RSFC between right medial orbitofrontal cortex and left amygdala correlated with response to lithium monotherapy in patients with bipolar disorder (75).

#### Dorsolateral Prefrontal Cortex

DLPFC, a large region spanning Brodmann Area 9 (BA9) and 46 (BA46) (88–90), plays a critical role in higher-order cognition and emotional processing. Numerous PET studies have shown decreased blood flow and oxygen consumption in depression (91), including some that specifically link cognitive symptoms of depression to DLPFC dysfunction (92, 93). Studies have predicted response to escitalopram and duloxetine with RSFC of DLPFC to bilateral middle frontal and inferior parietal regions (94).

As outlined earlier, a significant portion of recent seed-based analyses of DLPFC RSFC are in the context of TMS. Numerous studies have shown that DLPFC RSFC can correlate with or predict TMS response. In one study, higher RSFC between left DLPFC and striatum predicted TMS response (95). Several regions have been shown to exhibit connectivity changes after TMS response when DLPFC is the seed region, including parahippocampus (80) and left caudate (96). More studies are needed to clarify which downstream brain network nodes are most critical to TMS response.

#### Amygdala

As an integral node of the limbic system, amygdala has been most consistently implicated in response to negative stimuli in healthy controls. Several studies have identified altered amygdala function in depression and mania (18, 75, 97, 98), which likely inspired analyses of whether amygdala RSFC predicts or correlates with treatment response across various modalities. In early-life depression, increased baseline RSFC between amygdala, left DLPFC, and left anterior insula predicted treatment response to cognitive behavioral therapy (74). A similar strategy has been used to examine response to medications, although results are varied. In one study, increased baseline RSFC between amygdala, right central parietal opercular cortex, and Heschl's gyrus predicted response to fluoxetine or sertraline monotherapy in adolescents (99). Predictors of poor response to serotonergic medication for depression have been identified as increased baseline RSFC between amygdala, right precentral gyrus, and left supplementary motor area in one study (99), and increased baseline RSFC between amygdala and bilateral orbitofrontal cortex in a different study (94). Yet another study found that response to fluoxetine or sertraline was associated with increased RSFC between amygdala and right middle and middle frontal gyri, and decreased RSFC between amygdala and right posterior cingulate/precuneus (100). Limited sample sizes and methodological differences make it challenging to integrate these findings across studies.

Only one study investigating the impact of second-generation neuroleptic monotherapy on amygdala RSFC that met inclusion criteria. In this study, response to quetiapine was correlated with increased RSFC between left amygdala, superior and middle occipital gyri, and bilateral mid-cingulate, and between right amygdala and superior and middle occipital gyri and cuneus (101). This study was conducted in patients with unipolar depression and comorbid anxiety. Additionally, there was one study identifying the effect of a mood stabilizer on amygdala RSFC. In this study, increased RSFC between amygdala, rostral/subgenual ACC, and ventromedial prefrontal cortex correlated with improvements in depressive and hypomanic symptoms after lithium monotherapy (75).

There are some interesting studies implicating amygdala RSFC with response to real-time functional magnetic resonance imaging neurofeedback in patients with depression who are not taking medications (102, 103). One study demonstrated that abnormal RSFC between amygdala and several regions was reversed by real-time neurofeedback, with a specific emphasis on hippocampus (103). A different study showed that increased RSFC between amygdala and precuneus was associated with clinical improvement after real-time neurofeedback (102).

Amygdala RSFC does not appear to be thoroughly examined in the context of neuromodulation studies, but one study showed that patients with schizophrenia or major depressive disorder both showed significant RSFC decreases between right amygdala, right temporoparietal junction, medial prefrontal cortex, left posterior insula, and right DLPFC, and increases between right amygdala and hypothalamus after ECT. None of these changes correlated with changes in symptom severity (104).

#### Striatum

Striatum is thought to process several aspects of cognition in healthy controls, from motor planning to motivation and decision-making. In the context of mood disorders, psychomotor slowing or agitation have been linked to striatal connectivity changes (105). Nucleus accumbens, and ventral striatum more broadly, has been implicated in reward processed. Whereas, decreased RSFC has been linked to unipolar depression (106, 107), increased RSFC has been linked to bipolar disorder (108). One study showed that first episode mania was associated with decreased RSFC in the dorsal and caudal corticostriatal systems, and increased RSFC in the ventral striatal systems. Moreover, these baseline RSFC abnormalities predicted improvement in patients receiving lithium or quetiapine (109).

#### Insula

Insula is the one of the primary regions in which interoceptive information and emotional salience are processed (110, 111). Most of the mood disorder studies that have attempted to correlate insula RSFC to treatment response focus on psychotherapy. In one study, RSFC between right insula and right middle temporal gyrus predicted response to behavioral activation treatment in medication-free patients with unipolar depression (112). A similar study showed that successful cognitive behavioral therapy increased RSFC between right insula and left supragenual ACC in adolescents with unipolar depression (113).

One study of transdiagnostic cognitive behavioral therapy attempted to use baseline RSFC to predict improvement in emotional regulation rather than clinical outcomes. Several interesting results were generated by this study. At baseline, neuroticism was negatively correlated with RSFC between right dorsal anterior insula and inferior parietal lobule, and perception of impaired affective control was positively correlated with RSFC between ventral anterior insula and bilateral dorsal/pregenual ACC. Greater improvements in emotional regulation were predicted by decreased RSFC between right dorsal anterior insula and right ventrolateral prefrontal cortex as well as by increased RSFC between bilateral dorsal anterior insula and bilateral amygdala (114).

#### Hippocampus

Hippocampus plays critical roles in memory, cognition, and regulation of stress in healthy controls. It appears to be implicated in several RSFC analyses as a downstream node rather than the primary seed itself. One study showed that the increase in RSFC of right hippocampus after electroconvulsive therapy correlated with clinical improvement in elderly patients with varying degrees of unipolar depression (115).

#### Brainstem Nuclei

Aminergic nuclei have been hypothesized to play a role in mood disorders. One study found that patients treated with selective serotonin reuptake inhibitors had increased RSFC between dorsal raphe nucleus and precuneus, angular gyrus, and bilateral cerebellum, increased RSFC between locus coeruleus and occipital lobe, left precentral gyrus, and parahippocampal gyrus, and increased RSFC between ventral tegmental area and precuneus, left inferior parietal lobule, and bilateral middle/inferior temporal gyrus relative to patients treated with a serotonin norepinephrine reuptake inhibitor. By contrast, patients treated with a serotonin norepinephrine reuptake inhibitor had increased RSFC between dorsal raphe nucleus and right DLPFC, ventrolateral prefrontal cortex, and bilateral superior temporal cortex, increased RSFC between locus coeruleus and bilateral DLPFC, ventromedial prefrontal cortex, inferior temporal gyrus, and bilateral cerebellum, and increased RSFC between ventral tegmental area and left insula and bilateral cerebellum relative to the group treated with selective serotonin reuptake inhibitors (116).

In a separate study of young adults treated with selective serotonin reuptake inhibitors for unipolar depression, RSFC between ventral tegmental area and cuneus-occipital areas correlated with symptom improvement (117).

### Independent Component Analysis

Independent component analysis (ICA) is a statistical method used to discover hidden factors (components, sources, or features) in a set of measurements or observed data such that the factors are maximally independent. The main advantage of ICA is that it provides a data-driven means by which to measure whole-brain connectivity with all components considered.

Despite its strengths, ICA has a number of disadvantages and limitations. First, the process of identifying components and selecting methods with which to run ICA (e.g., dual regression) is subjective and variable. Second, inter-session reliability of component strength has not been fully established, which limits the degree to which ICA can reliably measure longitudinal treatment effects. Third, the functional attribution of each ICA component is indirectly assumed based on the brain regions included the analysis. Moreover, the function of those brain regions has been extrapolated from healthy controls in tasks that may or may not have translational significance. For example, the salience network is not consistently implicated across tasks that claim to test salience. As such, the functional significance of ICA components may change over time and between studies.

Several strategies have been proposed to address these imitations. One strategy involves examining the correlation between the time series extracted from each ICA component. This analysis would presumably measure connectivity between putative networks in a way that parallels seed-based analysis (118, 119). Unfortunately, the neurophysiological significance of the correlation between ICA components and the stability of this correlation within and between scanning sessions remain unclear, making it challenging to use these measures as treatment biomarkers. A different strategy involves reporting hypotheses and results in the form of ICA components even when the study used seed-based analyses, or vice versa (80, 120, 121).

ICA of RSFC has revealed several components comprised of correlated brain regions. These brain networks are named after their putative function in healthy controls, which provides some speculative basis for psychopathology (122, 123). In this review, ICA component terminology will only be used for studies in which an actual ICA analysis was conducted.

#### Default Mode Network

The default mode network is primarily comprised of the medial prefrontal cortex, ACC, posterior cingulate cortex, and angular gyrus. This intrinsic organizational structure shows high connectivity during wakeful rest and low connectivity during most goal-directed tasks, although there are some exceptions to these generalizations (123–125).

The default mode network has been implicated in clinical response across various treatment modalities, including psychotherapy, medications, and neuromodulation. In one study, responders to cognitive behavioral therapy or cognitive processing therapy had significantly higher increases in default mode network RSFC than non-responders (126).

Medication studies have shown varying results. In one study, an intravenous infusion of citalopram was correlated with positive RSFC between default mode network and left precuneus and negative RSFC between default mode network and amygdala in a group of patients with major depressive disorder. Interestingly, healthy controls who received the same infusion also had a positive correlation between default mode network and amygdala relative to healthy controls receiving a placebo infusion (127). In different studies, baseline RSFC of default mode network with orbitofrontal cortex was negatively correlated with improvement after 12 weeks of duloxetine (128).

Neuromodulation studies show results that may be consistent but are difficult to contextualize. In one TMS study, baseline RSFC between default mode network and ventromedial prefrontal cortex and ACC was more than 80% effective at discriminating responders from non-responders (119). In an ECT study, RSFC of default mode network to DLPFC was normalized after ECT response in late-life depression, and this increase in RSFC differentiated remitters from non-remitters (129).

#### Salience Network

The salience network, which is primarily comprised of insula and dorsal/pregenual ACC, is responsible for triaging stimuli and integrating multimodal information in healthy controls. As such, it is widely involved in communication, socialization, and self-monitoring (130). The salience network has most recently been implicated in TMS response. One study showed that baseline RSFC in the salience network was positively correlated with TMS treatment response (119). This result was replicated in a different study specifically examining early treatment response to TMS (131).

#### Inter-Network Connectivity

There are relatively few ICA studies of how inter-network connectivity may correlate with or predict treatment response in mood disorders. One study showed that higher baseline RSFC within the default mode network and between the default mode network and the central executive network predicted response to sertraline monotherapy in a relatively large sample of patients with unipolar depression (121).

#### Effective Connectivity

Effective connectivity is a means to infer causal or directional influences between brain network nodes (132). There were not many ICA studies examining treatment response in mood disorders. In one study, baseline fronto-insular effective connectivity was positively correlated with early response to TMS (131).

### Graph Theory Analysis

The application of graph theory to neuroimaging has yielded unique insights into network-wide properties rather than the strength of connectivity from a specific seed region (133). In this approach, a connection or adjacency matrix is used to summarize the nodes (brain regions) and edges (connections) of a brain network. A number of measures can be used to assess the matrix, including centrality (e.g., pageRank centrality, subgraph Centrality) assortativity (e.g., resilience), segregation (clustering coefficient, transitivity), and integration (e.g., diffusion efficiency).

The main advantage of graph theory is that it can provide a single variable for a network. As such, changes in that metric can be used to assess how an intervention affects the network. Despite this advantage, there are several limitations to consider First, the availability of numerous metrics can lead to numerous statistical analyses. Second, the stability of these metrics over time has not been established. Third, the significance of these metrics to putative function or clinical symptoms is unclear.

In this section, graph theory metrics are discussed in the context of treatment response in mood disorders.

#### Centrality

There are only a few studies examining a whole brain centrality measure in the context of mood disorder treatment. One study used eigenvector centrality to identify network nodes that are densely connected and sensitive to serotonergic medications. In this study of late-life depression, patients who remitted with venlafaxine showed significant RSFC increases between right precentral gyrus in the central executive network and significant decreases between right inferior frontal gyrus, supramarginal gyrus, and default mode network. Moreover, remitters showed significantly greater eigenvector centrality in bilateral inferior frontal gyrus and medial frontal gyrus than non-remitters (134). Using a slightly different metric, a study in patients with bipolar disorder showed that lithium treatment normalized mania-related connectome indices, reflected in part by significantly decreased right amygdala clustering coefficient (135).

One study assessed several metrics, including a centrality metric, to assess the effects of TMS on unipolar or bipolar depression. In this study, successful adjunctive TMS to dorsomedial prefrontal cortex resulted in significant increases in betweenness centrality in the stimulation target as well as in right amygdala, ventral striatum, and temporal pole. The authors noted that responders and non-responders showed opposing patterns of connectivity lateralization, and that patients with preserved hedonic function may be more responsive to dorsomedial TMS (136).

#### Assortativity

The study mentioned above that captured decreased right amygdala clustering coefficient with lithium treatment in bipolar disorder also assessed assortativity, which can be thought of as the degree to which a network node connects to similar nodes in a complex network. This study found that successful lithium treatment increased assortativity in a mood regulation network (135).

#### Global Brain Connectivity

One study leveraged the rapid-acting antidepressant effects of ketamine infusions to assess functional dysconnectivity changes in patients with major depression.

Responders to ketamine showed significant increases in global brain connectivity with global signal regression in lateral prefrontal cortex, caudate, and insula. The authors suggested that ketamine normalizes the dysconnectivity between these regions and the rest of the brain in major depressive disorder (137).

#### Connection Density

Connection density can be thought of as the number of observed connections relative to the number of possible connections in a graph or network. A few studies have examined this metric in the context of mood disorders treatment. The study of patients with bipolar disorder taking lithium that assessed clustering coefficient and assortativity also examined connection density. In this study, patients with bipolar disorder showed decreased mean connectivity in a network of which the largest percentage of differential links were with left posterior superior frontal gyrus, a midbrain region consisting of the red nucleus, substantia nigra, and ventral tegmental area, and right amygdala. Moreover, decreases in mania ratings were correlated with the decreases in in mean connectivity of this network (135).

A different study used network density and other measures to test the hypothesis that patients with dysthymic disorder have greater RSFC within the default mode network. At baseline, patients with dysthymic disorder showed higher default mode network RSFC than healthy participants, with specific elevations noted between posterior cingulate cortex and medial prefrontal cortex, bilateral lateral parietal lobes, and precuneus. After 10 weeks of duloxetine, patients with dysthymic disorder showed significantly reduced connectivity in many of these same connections. This “normalizing” effect was most prominent between posterior cingulate cortex, right lateral parietal cortex, and right inferior temporal gyrus (138).

### Other Methods

Aside from seed-based analysis, ICA, and graph theory analysis, several other methods have been developed to examine functional connectivity. A few examples that will not be covered here include regional homogeneity analysis (59, 139) and four-dimensional (spatiotemporal) consistency of local neural activities (FOCA) (140). These methods are not frequently used in treatment studies, and their functional significance and stability over time have not yet been established.

This section will briefly review coherence metrics, fractional amplitude of low-frequency fluctuation (fALFF), and machine learning.

#### Coherence Metrics

One study of patients with treatment-resistant depression receiving bilateral electroconvulsive therapy created maps of network coherence in each patient by using the mean time series of the default mode network (defined by ICA) as a regressor for each voxel within the default mode network. Maps from responders were compared to maps from non-responders and healthy controls using permutation testing. Patients with depression showed significantly decreased network coherence in precuneus and angular gyrus relative to healthy controls, and this difference normalized in electroconvulsive therapy responders but not non-responders. The authors interpreted this finding as preliminary evidence that electroconvulsive therapy reconnects a part of the default mode network to the broader network (141).

#### Fractional Amplitude of Low-Frequency Fluctuation

One of the primary goals of fALFF is to quantify the local, low frequency signals that often gets averaged across larger regions and frequency bands (59). This quantification is done by conducting a Fourier transformation on the BOLD signal and measuring power in ranges below 0.01 Hz. Several studies have employed fALFF to examine treatment effects of psychotherapy, medication, and neuromodulation for mood disorders.

A psychotherapy study used fALFF and other analyses to probe how cognitive remediation therapy changes intrinsic neural activity in patients with major depression. At baseline, patients with depression had reduced functional network strength in bilateral prefrontal systems. Intrinsic neural activity increased in right inferior frontal gyrus after cognitive remediation therapy, and activity changes in several areas including left inferior parietal lobule, left insula, left precuneus, and right caudate were associated with cognitive improvement (142).

A medication study used functional connectivity, effective connectivity, and fALFF to argue that major depression is associated with abnormal pulvinar oscillations and abnormal causal interactions between pulvinar and several nodes of default mode and posterior insular networks. They also show provide data that duloxetine can ameliorate this pulvinar pathophysiology (143).

A neuromodulation study used seed-based analysis and fALFF to probe the neurobiological substrates of electroconvulsive therapy response in unipolar or bipolar depression. At baseline, BOLD signal fluctuations (fALFF) in subcallosal cingulate cortex were significantly higher in patients with depression than they were in healthy controls. Successful electroconvulsive therapy significantly decreased these signal fluctuations (fALFF). Also, baseline signal fluctuation (fALFF) abnormalities predicted treatment response (144).

#### Machine Learning

Machine learning is a broad term that generally refers to the process of finding patterns in large, high dimensional datasets by training a computational model to predict unseen data. There are several ways to use machine learning to study treatment effects on brain networks. One study used a method called alternating decision trees to build models that accurately predicted late-life depression diagnosis and antidepressant treatment response with ~87 and 89% accuracy, respectively. Amongst other measures, these models included structural and functional connectivity. Lower RSFC of dorsal default mode network was specifically associated with positive treatment response (145).

A neuromodulation study leveraged RSFC and machine learning techniques to explore biomarkers of individual response to transcranial magnetic stimulation for depression. At baseline, patients with depression had low signal in caudate, prefrontal cortex, and thalamus. RSFC in default mode and affective networks was associated with treatment response. Using these findings, the authors successfully trained support vector machines to predict individual treatment response with 85–95% accuracy (146).

Machine learning has also been used to assess and predict individual response to electroconvulsive therapy. In one study, RSFC and multivariate pattern analysis identified a network centered in dorsomedial prefrontal cortex (including DLPFC, orbitofrontal cortex, and posterior cingulate cortex) that was 85% sensitive and 85% specific for individual response. A different network centered in the ACC (including DLPFC, sensorimotor cortex, parahippocampal gyrus, and midbrain) showed 80% sensitivity and 75% specific for individual response (147). A different study largely corroborated these results. In this study, a radial support vector machine was trained using arterial spin labeling and BOLD signal RSFC before electroconvulsive therapy for depression. The model predicted non-responders and responders with 74 and 64% accuracy, respectively, using connectivity strength among frontoparietal networks (including DLPFC), motor and temporal networks (near electroconvulsive therapy electrodes), and rostral/subgenual ACC (148).

## Discussion

### Summary

There are several reviews of task-based and RSFC biomarkers of mood disorders (46–55), but few evaluate evidence across diagnoses, models, and treatment modalities. In this review, we examined biomarker data categorized by analytic technique: (1) reference region (seed-based) analysis, (2) ICA, (3) graph theory analysis, and (4) other methods. This review supports our *a priori* hypothesis that there is no single mood disorder RSFC treatment biomarker validated across diagnoses, models, treatment modalities, and independent datasets.

### Reference Region (Seed-Based) Analysis: ACC, DLPFC, and Amygdala

Reference region (seed-based) analyses appear to be the most commonly used technique to assess RSFC biomarkers of treatment response in mood disorders. In some ways, it is the simplest and most direct way of discovering brain networks functionally connected to an *a priori* ROI (58, 149). Within this disproportionately large sample, ACC emerged as the region with the most consistent evidence across studies. This emergence has face validity given the well-established role of ACC as a nexus of cognitive (dorsal) and affective (ventral) processing (150–153). There are several lines of convergent evidence that Brodmann area 25 within the ACC plays a particularly critical role in mood regulation, driven in part by invasive and non-invasive neuromodulation. In TMS studies, stimulating DLPFC regions more functionally connected to Brodmann area 25 appears to enhance response (73, 154). This technique is emerging as the preferred targeting method in clinical trials (155, 156). Brodmann area 25 is also the most frequent target for deep brain stimulation, a controversial intervention for refractory depression with intriguing but mixed results (81–87). ACC also has unique cytoarchitecture (e.g., spindle cells or von Economo neurons) that could theoretically explain its role in mood disorders, but current imaging modalities have limited capacity to investigate this premise (157).

The other region that appears to emerge most consistently from reference region analyses is amygdala, which has structural and functional connections to prefrontal cortex, anterior cingulate, and other regions implicated in mood regulation. The role of amygdala has been identified previously (158, 159), but the current review emphasis its importance across diverse treatment modalities and contextualizes its role as both primary seed region and downstream network node for other seed regions. Interestingly, relatively few neuromodulation studies have focused on amygdala as a primary seed region.

### Independent Component Analysis: Default Mode Network

ICA appears to be the next most frequently used analysis technique for treatment biomarkers in mood disorders. This multivariate analysis avoids some of the biases and restrictions inherent to univariate reference region analysis (58, 160). Default mode network emerged as the construct with the most evidence across studies, which again implicates ACC as a critical node. It is difficult to evaluate default mode network as a biomarker of treatment response because it is so broadly implicated across tasks, non-tasks, and patient populations. Whereas, some studies report on connectivity exclusively within the default mode network, others reported on connectivity differences between individual nodes of the network and other network nodes that are not considered part of the network. There is also the possibility of the entire network correlating with another identified network, but this possibility raises several statistical and methodological challenges. It is also important to note that default mode network lacks specificity for mood disorders, further raising questions about how best to characterize it as a biomarker (161, 162).

### Machine Learning Analysis: Too Few Studies

There was no single region or network that emerged from graph theory analyses or other analyses, although these studies generally invoked individual network nodes identified with other analyses. A few machine learning studies were compelling in generating models with high predictive sensitivity and specificity, but these studies were in limited sample sizes and were not tested in independent datasets. They also typically lack causal evidence in the form of brain lesions or brain stimulation.

#### A Speculative Integration of Results

Despite the lack of consistent biomarker across diagnoses, models, treatment modalities, and independent datasets, the current results generally corroborate existing literature on the brain circuitry implicated in mood disorders. The DLPFC and ACC are frequently shown to be critical hubs in a network that mediates depressive symptoms. DLPFC lesions and blood flow changes correlated with depression motivated the earliest TMS studies (163, 164), and lesions with functional connectivity to this region are associated with depression (165). One of the main regions connected to DLPFC is Broadman area 25 (24) of the anterior cingulate cortex. Several studies have shown that activity in this region correlates with depressive symptoms (91, 166). From an explanatory model perspective, BA25 shows structural and functional connectivity to regions that could theoretically mediate depressive symptoms beyond low mood or sadness. Connections to medial and dorsolateral prefrontal cortex, orbitofrontal cortex, anterior, and posterior cingulate, amygdala, and hippocampus could mediate affective and executive symptoms, and connections to insula, hypothalamus, and monoaminergic brainstem nuclei could mediate neurovegetative symptoms (167). This theoretical model would encompass many of the regions and networks identified in this review.

One of several topics that needs to be further explored is the convergence and divergence between unipolar depression, bipolar depression, and mania. Presumably there is a region or network of regions that serves as a central regulator of mood, maintaining the balance between depression and mania as opposite ends of a mood spectrum, but more information is needed. There has been some recent progress in understanding mania as a state, although symptom specificity remains a challenge. For example, lesions associated with mania show a specific connectivity pattern that includes right orbitofrontal cortex, right inferior temporal gyrus, and right frontal pole (168). Future studies will continue to refine understanding of how mood is regulated throughout structurally or functionally connected brain networks.

### Limitations

There are several limitations that should be considered when interpreting the present results. First, the studies summarized in this review were heterogeneous in terms of patient populations, imaging acquisition quality or duration, analytic methods, and inter-scan reliability. It is beyond the scope of this review to outline these differences in fine detail, but nevertheless it is important to acknowledge that direct comparisons of potential biomarkers between heterogeneous studies should be done with caution. Second, the frequency with which a node or network is mentioned does not necessarily imply replication. For example, ACC could appear to be the region with the most evidence because the greatest number of studies chose it as a reference region or studied it in the context of the default mode network in ICA studies. The inverse problem applies to graph metric studies, which were fewer in number and thus difficult to contextualize. Third, this review does not address RSFC signal-to-noise or inter-scan reliability. RSFC is a dynamic measure, and studies could be using short scans that do not capture enough data (169). The dynamic nature of RSFC is particularly problematic for treatment effect studies that reply on repeated scans over time. There are ways to address these issues at the individual experiment level (170), but it is difficult to assess them in a review. Fourth, it is difficult to assess study design and implementation, particularly in terms of blind integrity and appropriate use of control participants. There are other limitations of this review, to say nothing of the construct validity of “mood disorders” as a category, but those previously discussed are some of the most basic ones to consider when interpreting the present results.

In order to evaluate candidate biomarkers, classification analyses should be run to assess the sensitivity and specificity in distinguishing responders from non-responders. This analysis should subsequently be validated in an independent dataset. A few studies have taken this approach, but the field at large is far from a validated RSFC biomarker of treatment response in mood disorders (118, 145, 146). It may also be the case that a single biomarker is unlikely to be successful because mood disorder are dynamic, heterogeneous, and multifactorial (171–173).

### Future Directions

Several strategies have been proposed to advance the study of candidate RSFC biomarkers and neuroimaging more broadly (174). One strategy focuses on individual- rather than group-level analyses, which could theoretically advance precision medicine in psychiatry as outlined by RDoC (5). This approach likely requires robust and repeated sampling from individuals over time, presenting both logistical and statistical challenges (174, 175). A seemingly opposite approach is to invest in larger samples that presumably enhance the power of data-driven analyses. This approach is evidenced by mega-analyses, mega-analyses, and a multitude of multi-site clinical projects such as Establishing Moderators and Biosignatures of Antidepressant Response in Clinical Care (EMBARC), International Study to Predict Optimized Treatment in Depression (iSPOT-D), The Predictors of Remission in Depression and Individual and Combined Treatment (PReDICT), Response to Lithium Network (R-LiNK), and others (176, 177). Larger datasets may increase statistical power, but they also potentially compound noise and variables.

A third approach involves transdiagnostic studies, which presumably avoids the assumptions of searching for neurobiological correlates of symptom clusters that lack biological validity. Examples of this approach typically focus on the “p factor” of general psychopathology, with the long-term strategy of potentially reverse engineering clinical constructs based on brain networks rather than symptom clusters (178–180). This long-term strategy has a number of challenges and is particularly difficult to implement with respect to treatment response.

A fourth strategy is to study focal brain lesions or brain stimulation to assess causality in networks that are potential biomarkers (181, 182). Distinguishing correlation from causation is challenging in traditional neuroimaging studies because network changes may be a cause of, an effect of, or an adaptation to a mood disorder or its treatment (21, 181). A computational technique called network mapping leverages the statistical power of the human connectome (183) to map atrophy coordinates, lesions, or stimulation sites to whole-brain networks rather than single brain regions (21). This technique has been used to identify new neuromodulation treatment targets, and to optimize existing neuromodulation treatment targets for neuropsychiatric conditions (154, 184). Network mapping is powerful, but it also has several limitations as a retrospective meta-analytic technique using normative connectome data to examine symptoms caused by lesions. It is also challenging to assess causality with lesions because brain disorders, like most disorders in medicine, have biopsychosocial aspects to them.

## Conclusions

Many disparate findings have been reported for RSFC biomarkers of treatment response in mood disorders. These findings are complicated by small sample sizes, potential biases, and study heterogeneity. As such, no single biomarker has been identified or validated across diagnoses, models, or treatment modalities. Despite these current limitations, there are several future direction that could facilitate the identification of treatment response biomarkers in mood disorders.

## Author Contributions

All authors contributed to the article and approved the submitted version.

## Conflict of Interest

The authors declare that the research was conducted in the absence of any commercial or financial relationships that could be construed as a potential conflict of interest.
